# Supplementary material for: The Apparent Organ-Specificity of Amyloidogenic ApoA-I Variants Is Linked to Tissue-Specific Extracellular Matrix Components
Source: Int J Mol Sci. 2022 Dec 24;24(1):318. doi: 10.3390/ijms24010318 (PMC9820410; doi:10.3390/ijms24010318)
Supplement: Supplementary file 1 [file ijms-24-00318-s001.zip › ijms-2035307-supplementary.pdf]

Supplementary material to:

The apparent organ-specificity of amyloidogenic ApoA-I variants is linked to tissue-specific extracellular matrix components

Rita Del Giudice [1,#,*], Mikaela Lindvall [1], Oktawia Nilsson [1], Daria Maria Monti [2,3] and Jens O Lagerstedt [1,4,§,*]

[1] Department of Experimental Medical Science, Lund University, 221 84 Lund, Sweden; R.D.G: rita.del-giudice@mau.se; M.L.: mika.lindvall@gmail.com; O.N.: oktawia.nilsson@gmail.com
[2] Department of Chemical Sciences, University of Napoli Federico II, Complesso Universitario Monte Sant'Angelo, 80126 Napoli, Italy mdmonti@unina.it
[3] Istituto Nazionale di Biostrutture e Biosistemi (INBB), 00136 Rome, Italy
[4] Islet Cell Exocytosis, Department of Clinical Sciences Malmö, Lund University Diabetes Centre, Lund University, Malmö, Sweden jens.lagerstedt@med.lu.se
[#] Current address: Department of Biomedical Science and Biofilms — Research Center for Biointerfaces, Malmö University, 20506 Malmö, Sweden
[§] Current address: Novo Nordisk A/S, Copenhagen, Denmark
* Correspondence: R.D.G. rita.del-giudice@mau.se; J.O.L. jens.lagerstedt@med.lu.se

Data included in this file:

Figure S1. ApoA-I amyloidogenic variants affect cell viability in a time and dose-dependent manner
Figure S2. Lipidation attenuates the toxicity of ApoA-I amyloidogenic variants
Figure S3. ApoA-I variants' cytotoxic effect is not due to altered redox homeostasis
Figure S4: Cellular localization of ApoA-I amyloidogenic variants in HaCaT keratinocytes
Figure S5: Cellular localization of ApoA-I amyloidogenic variants in HepG2 hepatocytes
Figure S6. Comparison between the binding to heparin of ApoA-I proteins in the lipid free and rHDL-bound forms
Figure S7. Secondary structure estimation of ApoA-I amyloidogenic variants in the presence of the different ECM components at physiological pH
Figure S8. Synchrotron Radiation Circular Dichroism spectra of ApoA-I amyloidogenic variants in the presence of the different ECM components at pathophysiological pH

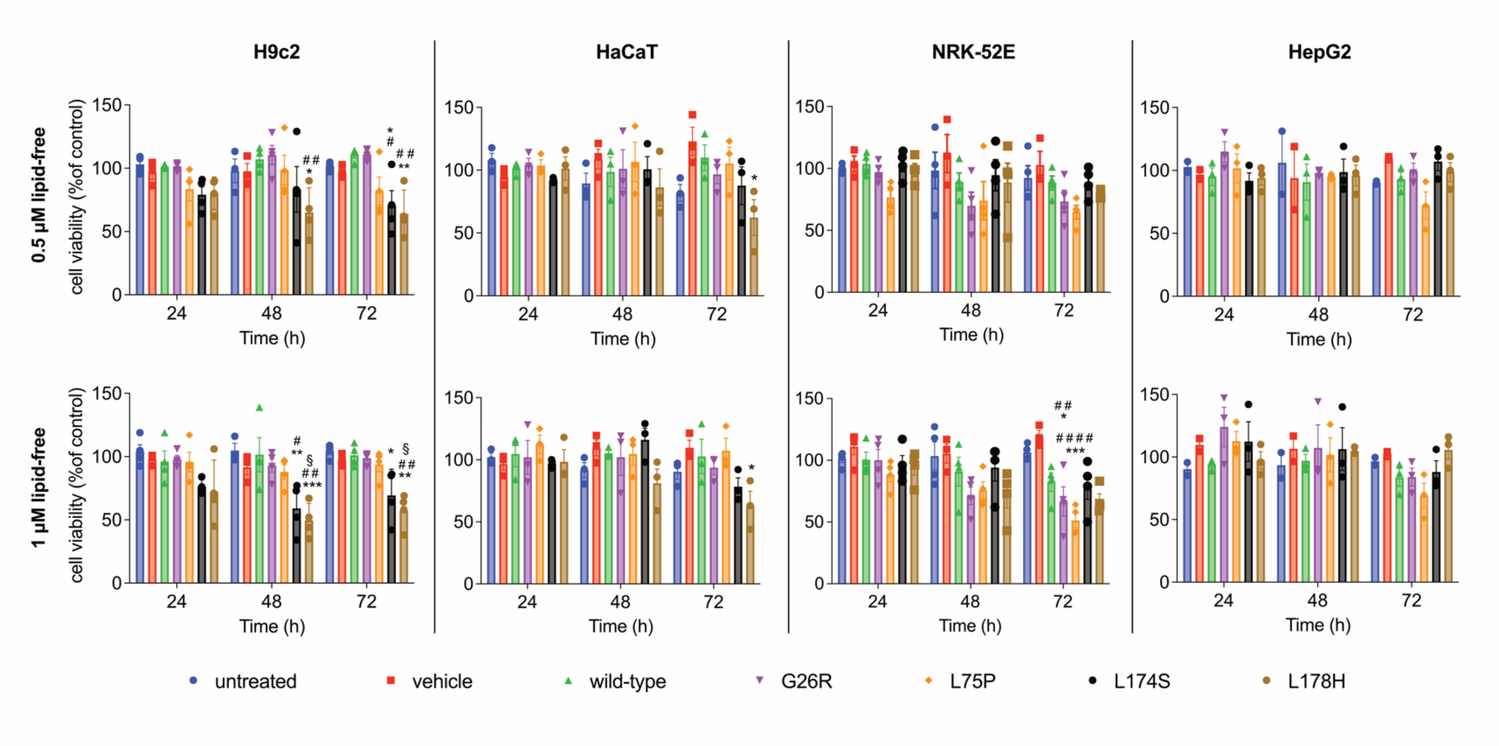

Figure S1: ApoA-I amyloidogenic variants affect cell viability in a time and dose-dependent manner. H9c2, NRK-52E, HaCaT and HepG2 cells were incubated with 0.5 and 1 μM of each ApoA-I protein for the indicated length of time, and cell viability was determined by a MTT (3-[4,5-dimethylthiazol-2-yl]-2,5-diphenyltetrazolium bromide) assay. Data shown are the means ± SEM of independent experiments (n = two to four) carried out in triplicate. Significance was calculated according to 2way ANOVA (*p < 0.05, **p < 0.005, ***p < 0.001, for groups as indicated with respect to untreated cells, #p < 0.05, ##p < 0.005, ####p < 0.0001 refers to groups as indicated with respect to vehicle treated cells, §p < 0.05 for groups as indicated with respect to the WT protein).

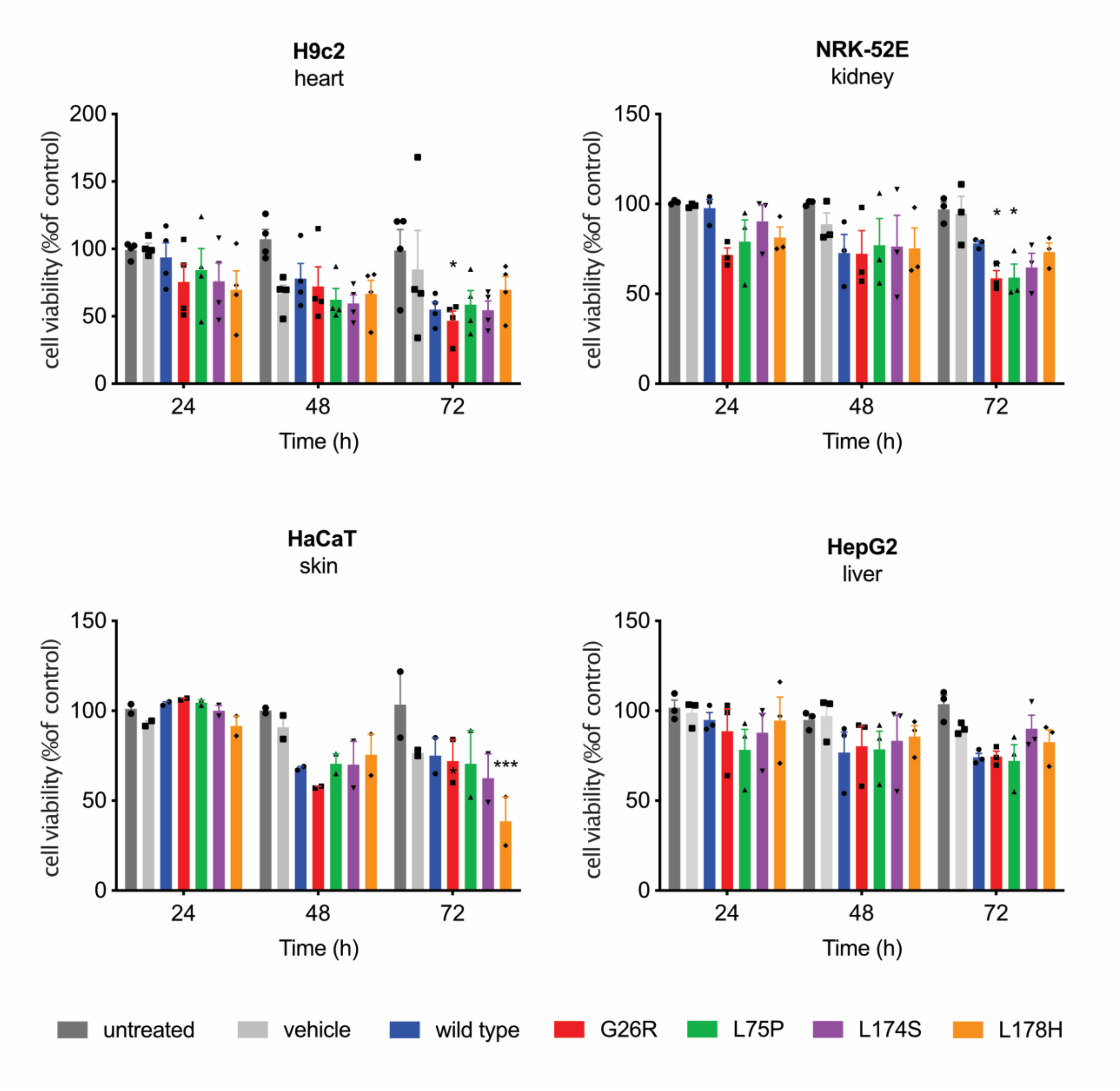

Figure S2. Lipidation attenuates the toxicity of ApoA-I amyloidogenic variants. H9c2, NRK-52E, HaCat and HepG2 cells were incubated with 2 µM of each ApoA-I amyloidogenic variant, as well as the WT protein, in DMPC reconstituted HDL, for the indicated length of time. At the end of incubation, MTT (3-[4,5-dimethylthiazol-2-yl]-2,5- diphenyltetrazolium bromide) was performed and cell viability determined. Data shown are the means ± SEM of two to four independent experiments carried out in triplicate. Significance was calculated according to two-way ANOVA ***$p < 0.001$ with respect to untreated cells

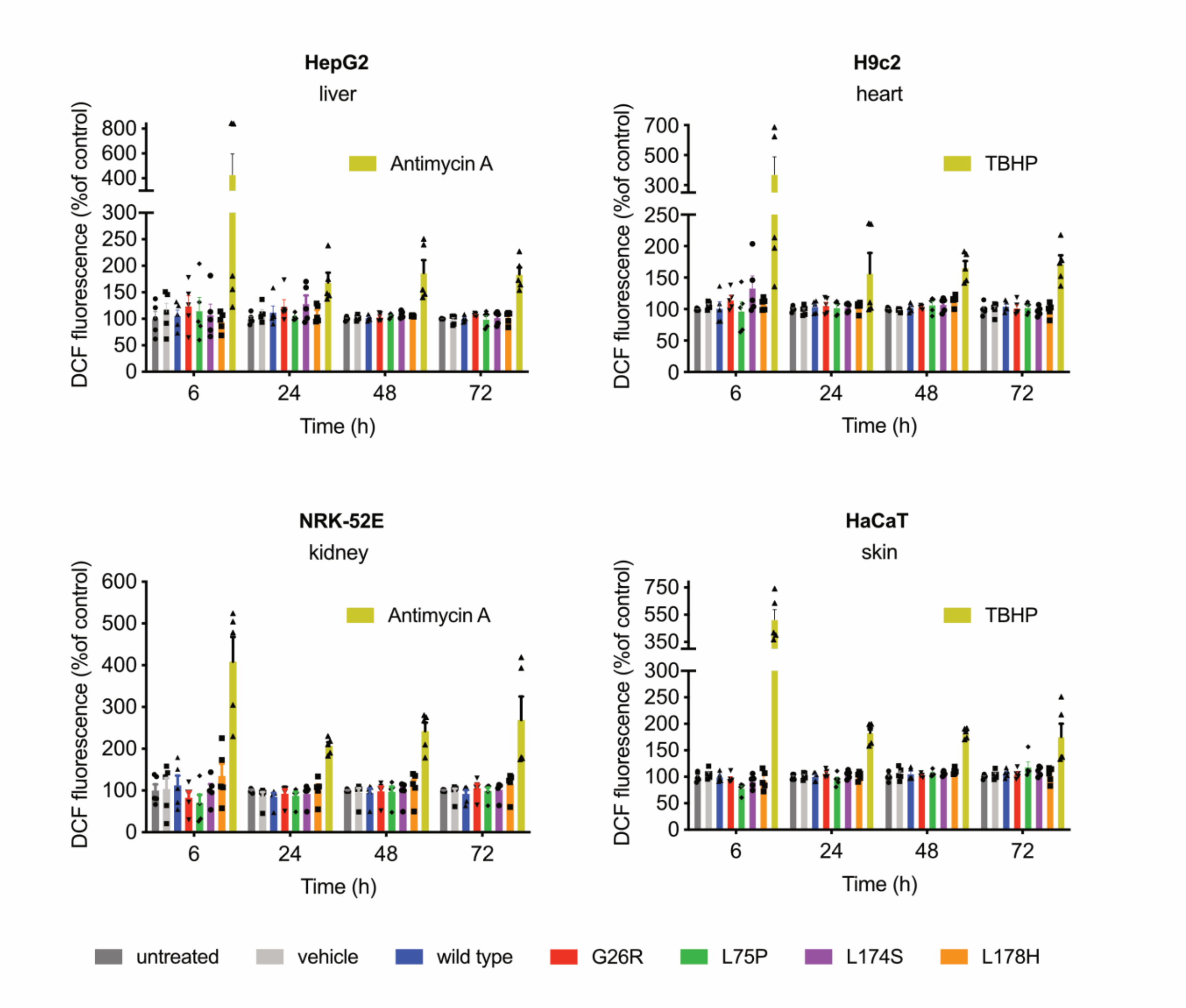

Figure S3. ApoA-I variants' cytotoxic effect is not due to altered redox homeostasis. H9c2, NRK-52E, HaCat and HepG2 cells were incubated with 2 μM of each ApoA-I protein, as well as with 1 mM of antimycin A or 50 μM of TBHP, for the indicated length of time. At the end of the incubation, reactive oxygen species levels were measured by evaluating the fluorescence of DCF. Data shown are the means ± SEM of five independent experiments carried out in duplicate. Significance was calculated according to two-way ANOVA.

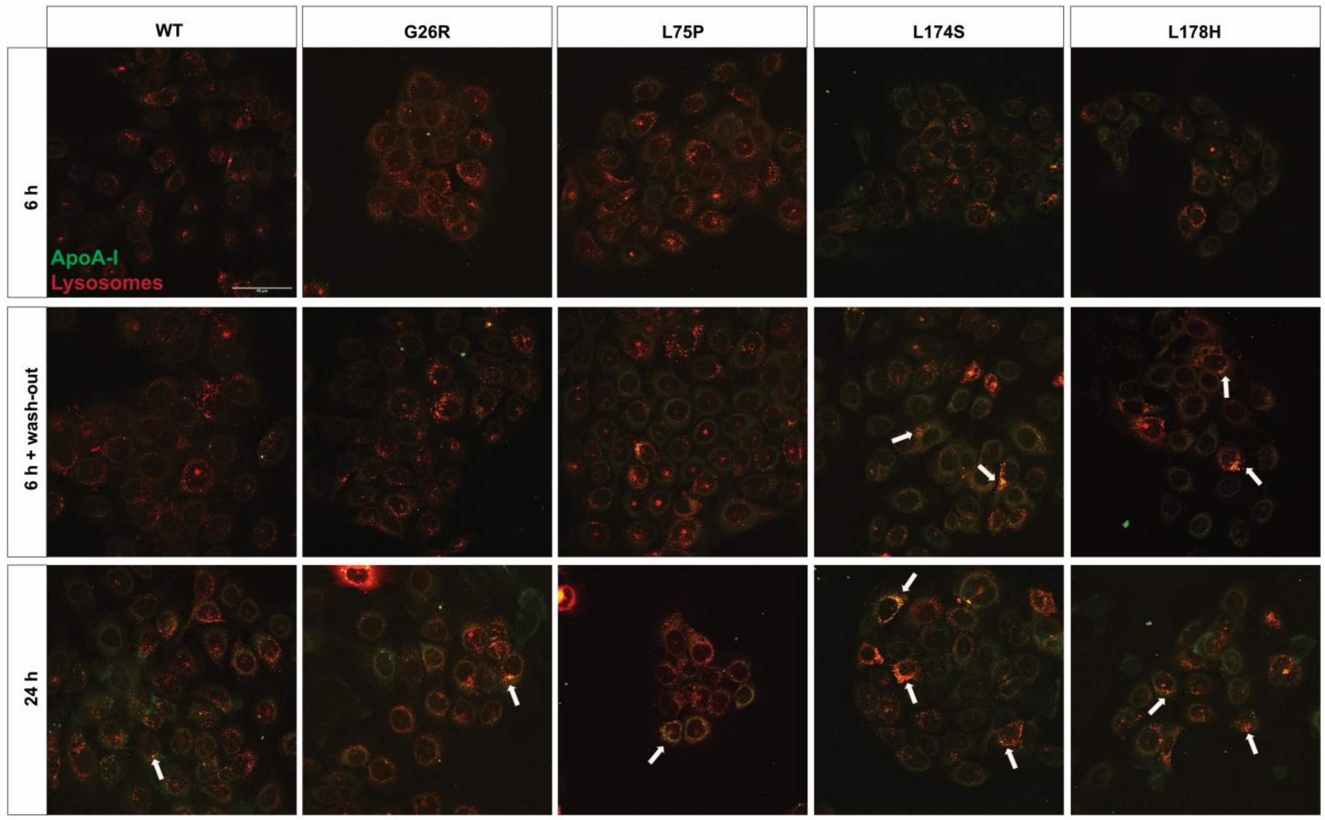

Figure S4. Cellular localization of ApoA-I amyloidogenic variants in HaCaT keratinocytes. HaCaT cells were incubated at 37°C with 2 μM FITC-labelled ApoA-I proteins (green) for 6 h (upper and middle panels) or 24 h (lower panels). To study the ability of the lysosomes to degrade the ApoA-I proteins, cells incubated with FITC-ApoA-I for 6 h were incubated for additional 16 h in absence of the labelled protein (6 h + wash-out, middle panel). Lysosomes were stained with Lysotracker red. Cells were imaged by confocal microscopy using a 60x objective. Scale bar is 50 μm. White arrows indicate protein co-compartmentalization with the lysosomes.

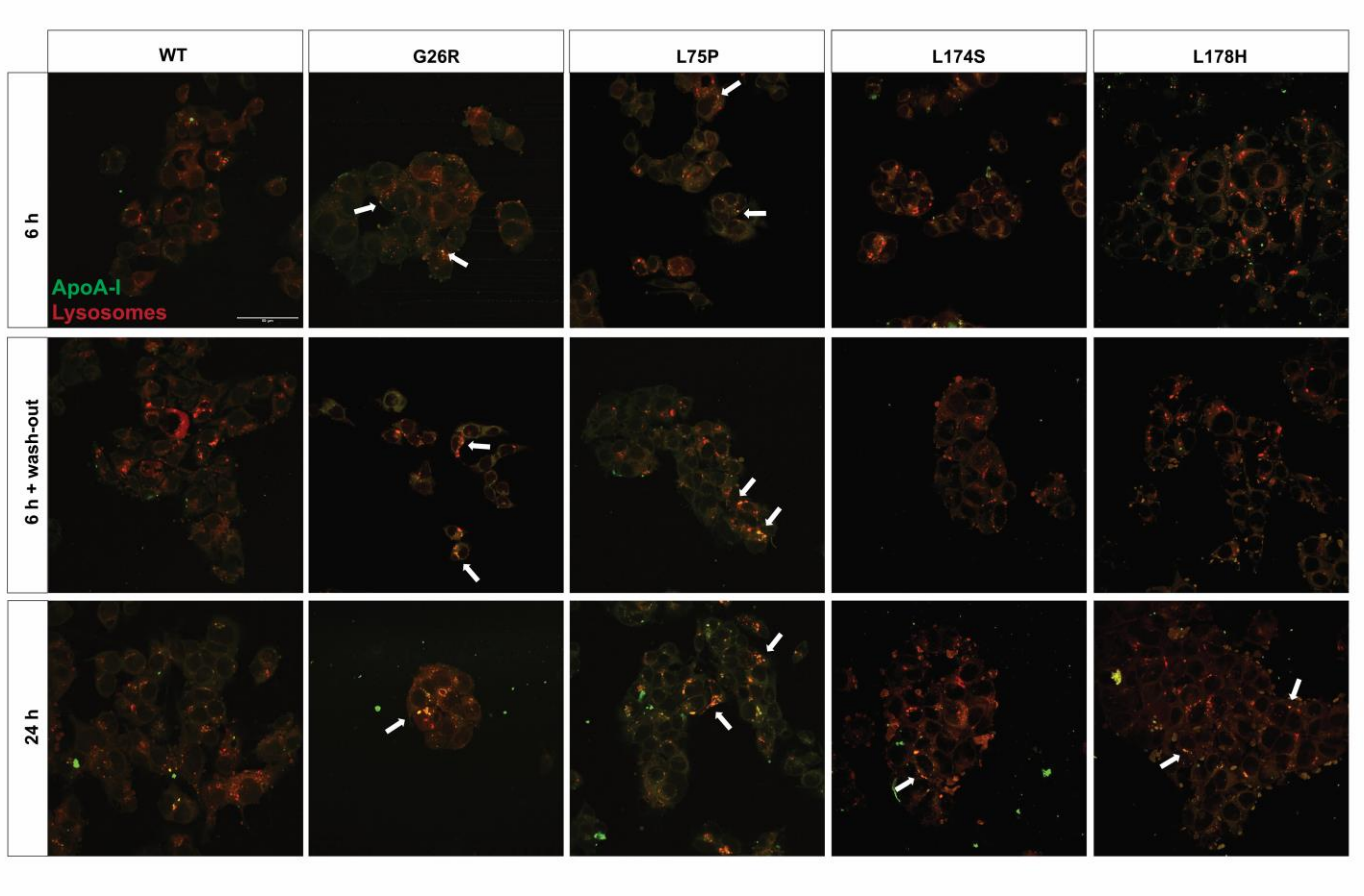

Figure S5. Cellular localization of ApoA-I amyloidogenic variants in HepG2 hepatocytes. HepG2 cells were incubated at 37°C with 2 μM FITC-labelled ApoA-I proteins (green) for 6 h (upper and middle panels) or 24 h (lower panels). To study the ability of the lysosomes to degrade the ApoA-I proteins, cells incubated with FITC-ApoA-I for 6 h were incubated for additional 16 h in absence of the labelled protein (6 h + wash-out, middle panel). Lysosomes were stained with Lysotracker red. Cells were imaged by confocal microscopy using a 60x objective. Scale bar is 50 μm. White arrows indicate protein co-compartmentalization with the lysosomes.

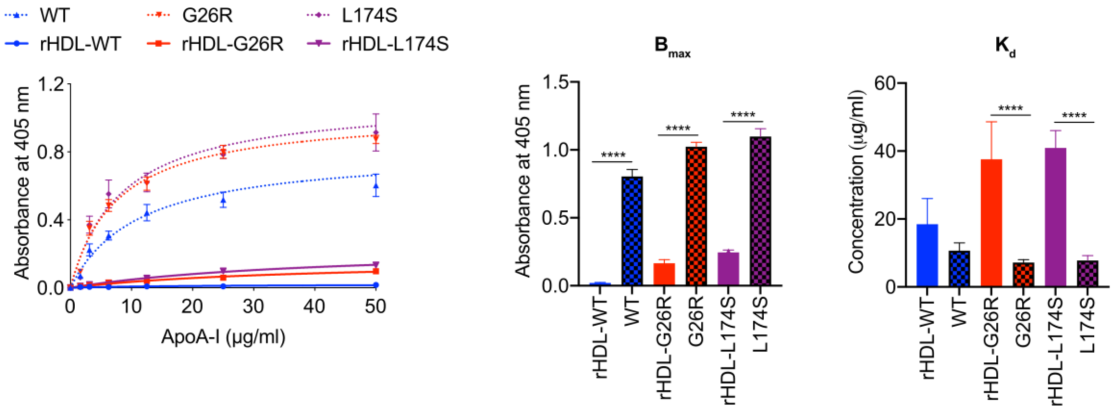

Figure S6. Comparison between the binding to heparin of ApoA-I proteins in the lipid free and rHDL-bound forms. **Left panel**, Binding to heparin quantified as a function of the concentration of lipid free (dashed line) or rHDL-bound (continuous line) ApoA-I proteins (left panel). Comparison of the B_{max} (middle panel) and K_d values (right panel) calculated for the lipid free (pattern-filled bars) and rHDL-bound (color-filled bars). Significance is calculated according to one-way ANOVA (****$p < 0.0001$)

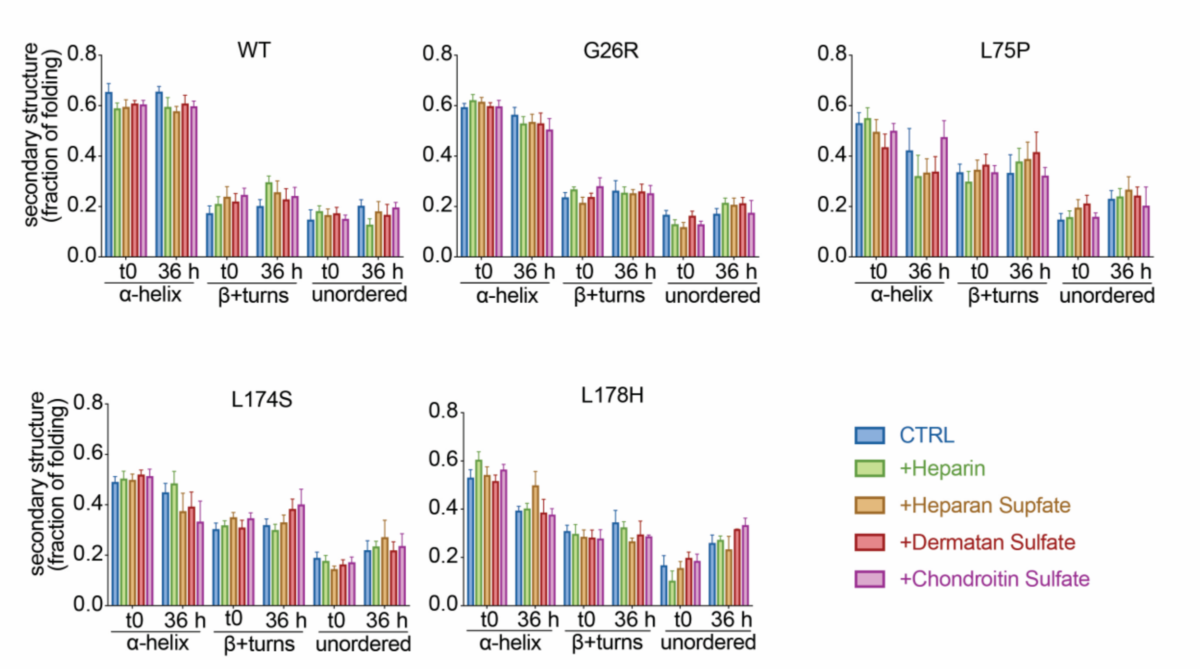

Figure S7. Secondary structure estimation of ApoA-I amyloidogenic variants in the presence of the different ECM components at physiological pH. ApoA-I amyloidogenic variants as well as the WT protein were incubated in McIlvaine buffer at pH 7.4, at 37°C in the presence of the different ECM components at a protein concentration of 0.5 mg/ml and a protein to ECM component molar ratio of 1:2. At the indicated time points, samples were analyzed by SRCD, and fraction of protein secondary structure elements obtained by deconvoluting SRCD spectra with the CDSSTR algorithm. Data shown are the mean ± SEM of at least 3 independent experiments carried out in duplicate.

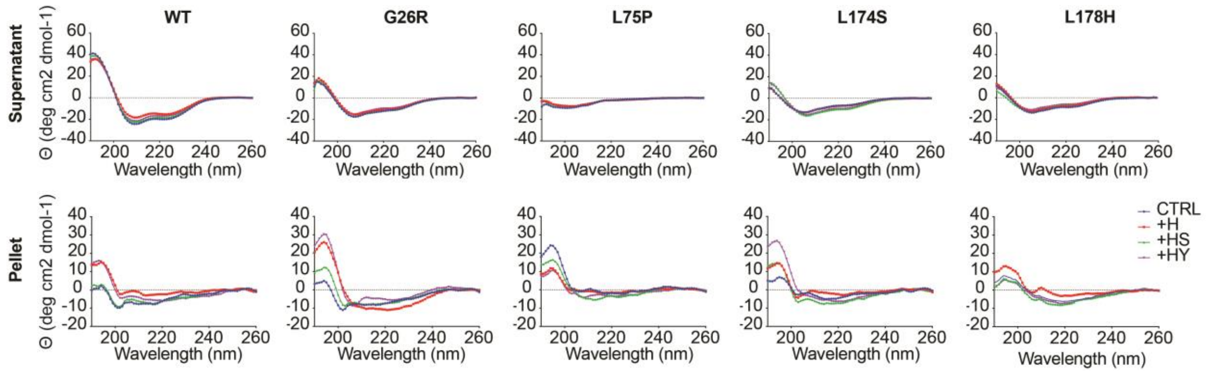

Figure S8. Synchrotron Radiation Circular Dichroism spectra of ApoA-I amyloidogenic variants in the presence of the different ECM components at pathophysiological pH. ApoA-I amyloidogenic variants (2 mg/ml), as well as the native protein, were incubated with the different ECM components (1:2 protein to ECM molar ratio) for one week in McIlvain buffer, pH 6.4, at 2 mg/mL, under agitation. At the end of the incubation, samples were centrifuged at 20,000 x g for 30 minutes at 4 °C and SRCD spectra acquired for both the supernatant and the insoluble protein samples.
